# Genome-wide association study-identified SNPs (rs3790844, rs3790843) in the *NR5A2* gene and risk of pancreatic cancer in Japanese

**DOI:** 10.1038/srep17018

**Published:** 2015-11-23

**Authors:** Makoto Ueno, Shinichi Ohkawa, Manabu Morimoto, Hiroshi Ishii, Masato Matsuyama, Sawako Kuruma, Naoto Egawa, Haruhisa Nakao, Mitsuru Mori, Keitaro Matsuo, Satoyo Hosono, Masanori Nojima, Kenji Wakai, Kozue Nakamura, Akiko Tamakoshi, Mami Takahashi, Kazuaki Shimada, Takeshi Nishiyama, Shogo Kikuchi, Yingsong Lin

**Affiliations:** 1 Hepatobiliary and Pancreatic Medical Oncology Division, Kanagawa Cancer Center Hospital, Kanagawa 241-8515, Japan; 2 Hepatobiliary and Pancreatic Section, Gastroenterological Division, Cancer Institute Hospital, Tokyo 135-8550, Japan; 3 Department of Internal Medicine, Tokyo Metropolitan Komagome Hospital, Tokyo 113-8677, Japan; 4 Tokyo Metropolitan Ohtsuka Hospital, Tokyo 170-8476, Japan; 5 Division of Gastroenterology, Department of Internal Medicine, Aichi Medical University School of Medicine, Nagakute 480-1195, Japan; 6 Department of Public Health, Sapporo Medical University School of Medicine, Sapporo 060-0061, Japan; 7 Division of Molecular Medicine, Aichi Cancer Center Research Insitute, Nagoya 762-6111, Japan; 8 Department of Epidemiology, Nagoya University Graduate School of Medicine, Nagoya 466-8550, Japan; 9 Division of Epidemiology and Prevention, Aichi Cancer Center Research Institute, Nagoya 762-6111, Japan; 10 Department of Preventive Medicine, Nagoya University Graduate School of Medicine, Nagoya 466-8550, Japan; 11 Department of Food and Nutrition, Gifu City Women’s College, Gifu 501-2592, Japan; 12 Department of Public Health, Hokkaido University Graduate School of Medicine, Sapporo 060-8638, Japan; 13 Central Animal Division, National Cancer Center Research Institute, Tokyo 104-0045, Japan; 14 Department of Hepatobiliary and Pancreatic Surgery, National Cancer Center Hospital, Tokyo 104-0045, Japan; 15 Department of Public Health, Aichi Medical University School of Medicine, Nagakute 480-1195, Japan

## Abstract

We genotyped 2 SNPs (rs3790844 T/C and rs3790843 G/A) in the *NR5A2* gene that were identified in a genome-wide association study (GWAS) of pancreatic cancer in populations of mainly European ancestry, and we examined their associations with pancreatic cancer risk in a case-control study of 360 patients and 400 control subjects in Japan. Unconditional logistic regression models were used to estimate odds ratios (ORs) and 95% confidence intervals (CIs). The SNPs were in linkage disequilibrium (r^2^ = 0.80). For rs3790843, the multivariable-adjusted OR was 0.75 (95% CI: 0.41–1.36) and 0.60 (95%CI: 0.33–1.08) for subjects with the AG and AA genotype, respectively, compared to subjects with the GG genotype. The per allele OR was 0.78 (0.62–0.99) (P = 0.046). For rs3790844, the multivariable-adjusted OR was 0.65 (95% CI: 0.37–1.14) and 0.47 (95%CI: 0.27–0.83) for subjects with the CT and CC genotype, respectively, compared to subjects with the TT genotype. The per allele OR was 0.70 (0.56–0.89) (P = 0.003). Our case-control study found that rs3790843 and rs3790844 in the *NR5A2* gene are associated with pancreatic cancer risk in Japanese subjects. The direction of association is consistent with the prior findings from GWASs.

Pancreatic cancer is the fifth leading cause of cancer death in Japan, accounting for approximately 30,000 deaths annually. The incidence and mortality have shown an increasing trend over the past decades^1^. Pancreatic cancer is often diagnosed at an advanced stage and rapidly fatal, with a 5 year survival rate of less than 10%^2^. Much is still unknown about the etiology of pancreatic cancer. Although smoking and longstanding diabetes are two well-established risk factors, they accounted for only a small proportion of pancreatic cancer^3^. The role of genetic influences is important, with increasing evidence supporting the notion that common genetic variants in multiple genes may act collectively to confer susceptibility to pancreatic cancer^4^.

Genome-wide association studies (GWASs) are a powerful, hypothesis-free approach that can identify inherited genetic variations associated with human traits and disease risk^5^. Since their inception, GWASs have illuminated novel pathways involved in the development and progression of various cancer types^6^. For pancreatic cancer, 5 GWASs conducted in populations of European, Japanese, and Chinese ancestry have reported 17 loci associated with increased susceptibility^7^,^8^,^9^,^10^,^11^. The first GWAS for pancreatic cancer identified common risk variants that map to the first intron of the ABO gene on chromosome 9q34.2^7^, corroborating earlier epidemiologic findings showing a lower risk among individuals with blood group O than those with blood group A or B^12^,^13^. The association of ABO loci with pancreatic cancer has been robustly replicated in subsequent studies of diverse ethnic populations^12^,^14^,^15^,^16^, including Chinese^12^ and Japanese populations^15^. In the second GWAS for pancreatic cancer, three loci, on chromosomes 13q22.1, 1q32.1 and 5p15.33, were found to be associated with pancreatic cancer susceptibility^8^. Of the 5 highly significant single nucleotide polymorphisms (SNPs) on 1q32.1 that map to *NR5A2*, rs3790844 showed the strongest association with pancreatic cancer, with a per-allele OR of 0.77 (95% CI: 0.71–0.84). rs3790843 was highly correlated with rs3790844 in the Pancreatic Cancer Consortium (PanScan) European controls (r^2^ = 0.59)^8^. *NR5A2* encodes a nuclear receptor of the fushi tarazu subfamily and is involved in development, and cholesterol and bile-acid homeostasis^17^. It is notable that two recent function studies have highlighted a crucial role of *NR5A2* in the homeostasis of pancreatic acinar differentiation and in inflammation^18^,^19^. A subsequent GWAS reported additional loci that associated with risk of pancreatic cancer; however, the function of the identified variants needed to be defined in further studies^9^,^10^,^11^.

Efforts are underway to replicate GWASs-identified SNPs in different ethnic populations^12^,^14^,^15^,^16^,^20^,^21^,^22^,^23^. Although rs3790843 and rs3790844 in the *NR5A2* gene have been replicated in a previous study involving subjects of mainly European ancestry^21^, it remains unclear whether these 2 SNPs are also associated with pancreatic cancer risk in Asian populations. Given the possible differences in minor allele frequency and pattern of linkage disequilibrium between Asian and Western populations, genetic susceptibility to pancreatic cancer may vary. We therefore examined the association between 2 SNPs (rs3790843 and rs3790844) in the *NR5A2* gene and the risk of pancreatic cancer using data collected from a genetic case-control association study of Japanese subjects.

## Results

The distribution of genotypes among the control subjects did not deviate from HWE (P = 0.64 for rs3790843 and P = 0.54 for rs3790844). Table 1 shows the selected characteristics of the cases and control subjects. The mean age was 67.8 ± 8.8 years for the cases and 64.8 ± 9.5 years for the control subjects. The proportion of subjects who had a history of diabetes was higher in the cases than in the control subjects (24.1% versus 8.7%, respectively). The current smokers represented 59.7% in the cases and 49.5% in the control subjects. The proportion of case patients with tumor stage I, II, III and IV was 4.2%, 15.0%, 21.4% and 58.3%, respectively. The majority of case patients were diagnosed at an advanced stage.

The association between rs3790843 or rs3790844 in the NR5A2 gene and risk of pancreatic cancer is shown in Table 2. For rs3790843, the subjects with an AA genotype had a decreased risk, with a multivariable-adjusted OR of 0.60 (95% CI: 0.27–1.08). The per allele OR was 0.78 (95% CI: 0.62–0.99) (P = 0.046). rs3790844 was significantly associated with pancreatic cancer risk; the OR was 0.47 (95% CI: 0.27–0.83) for the subjects with a CC genotype compared with the subjects with a TT genotype. The per allele OR was 0.70 (95% CI: 0.56–0.89) (P = 0.003). Table 3 shows the associations between two SNPs and risk of pancreatic cancer according to BMI. For 3790843, in either group, the AA genotype was associated with a decreased risk compared with the GG genotype. For rs3790844, in either group, the CC genotype was associated with a decreased risk compared with the TT genotype.

False positive report probability (FPRP) was calculated to assess whether significant findings were “noteworthy”. Results of FPRP calculations based on different prior probability and ORs were provided in Tables S1 and S2 as Supplementary information. For rs3790844, the obtained FPRP value was 0.06 if the prespecified FPRP value was set at 0.2 and the prior probability at 0.1 (Table S1). For rs3790843, the obtained FPRP value was 0.47 if the prespecified FPRP value was set at 0.2 and the prior probability at 0.1 (Table S2). These findings suggested that the positive result for rs3790844 was more “noteworthy”.

## Discussion

In this genetic case-control association study, we genotyped 2 GWASs-identified SNPs (rs3790843 and rs3790844) in the *NR5A2* gene and examined their association with pancreatic cancer risk. We found that they were also associated with pancreatic cancer risk in these Japanese subjects. The calculation of FPRP showed that significant findings on these two SNPs were noteworthy. Furthermore, BMI did not modify the association between two SNPs and pancreatic cancer risk.

The replication of GWAS-identified SNPs in populations of non-European ancestry is important because successful replication can increase the credibility of the original association; however, studies replicating GWASs-identified variants across populations have yielded mixed results. For example, a previous study involving individuals of European ancestry successfully replicated the major loci reported in the original GWAS involving individuals of European ancestry, whereas it failed to replicate the association of pancreatic cancer with 7 GWASs-identified SNPs in two Asian populations^22^. Two previous studies have addressed the association between *NR5A2* genetic variants and pancreatic cancer risk^21^,^22^, but the results were not entirely consistent. One case-control study examined genetic variants and pancreatic cancer risk in populations of mainly non-Hispanic whites in the United States and replicated three SNPs (rs3790843, rs3790844 and rs12029406) that were identified in the original GWAS^21^. For all three SNPs, the minor homozygotes were associated with a decreased risk of developing pancreatic cancer compared with the major homozygotes. By contrast, the other case-control study involving European populations did not show a significant association between rs3790844 and pancreatic cancer risk^22^ but found that rs12029406 in the *NR5A2* gene was associated with pancreatic cancer prognosis. Possible reasons for the inconsistent findings in replicating GWASs-identified SNPs include the differences in allele frequencies and patterns of linkage disequilibrium (LD) or hidden effects of gene-gene or gene-environment interaction across populations^24^. It is notable that we found a marked allele-frequency difference in rs3790844 between European and Japanese populations; the minor allele C of rs3790844 among the control subjects in the original GWAS was 0.24, whereas it was 0.73 in this study. Despite the allele-frequency differences, the association observed in this study was in the same direction with that observed in the original GWAS, and the per-allele was comparable between our study and the original GWAS (0.70 versus 0.77); however, whether the allele-frequency differences are due to genetic drift or natural selection remains to be elucidated. As for patterns of linkage disequilibrium, the r^2^ was 0.80 between rs3790843 and rs3790844 in our samples, which was higher compared with that (r^2^ = 0.59) reported in the prior GWAS^8^.

Because there has been evidence showing that obesity-related FTO gene variants were associated with pancreatic cancer only in overweight people^21^, we conducted additional analyses examining a possible effect modification by BMI. Our findings suggested that BMI did not modify the association between rs3790844 and pancreatic cancer risk.

The SNP rs3790844 is located in the first intron of the *NR5A2* gene, which encodes a nuclear receptor that participates in a wide range of developmental processes^17^. In addition, *NR5A2* regulates cholesterol, fatty acids and bile acid homeostasis^17^. Available evidence suggested that alterations in *NR5A2* function might promote pancreatic carcinogenesis^18^,^19^. A recent study showed that *NR5A2* plays a critical role in maintaining acinar cell differentiation^18^. Loss of acinar *NR5A2* was found to accelerate the development of oncogenic *Kras* driven acinar to ductal metaplasia and pancreatic cancer precursor lesions^18^. Because rs3790844 is located in a non-coding region, how the variations at this location influence *NR5A2* gene function or expression level is not clear. Future studies will be required to address this issue.

Our study has several limitations. First, our sample size was relatively small, although we were able to replicate the GWAS-identified SNP in this study. Another limitation inherent in a case-control study is that the selection of control subjects is prone to bias; however, bias due to control selection is generally not a problem for genetic case-control association studies. Instead, population stratification, which relates to the ethnic origin of cases and controls, is a major issue worth addressing because it can cause spurious association^25^. A previous study of Japanese population structure has clearly demonstrated that most Japanese individuals fall into two clusters: the Hondo cluster and the Ryukyu cluster. The vast majority of subjects in our study belong to the Hondo cluster, so we consider that population stratification may not be a problem. In addition, the allele frequencies (C: 0.73; T: 0.27) observed in our control subjects were similar to those (C: 0.65; T: 0.33) reported for Japanese subjects in the HapMap Project (accessed athttp:// www.ncbi.nlm.nih.gov/projects/SNP/snp_ref.cgi? rs=3790844), suggesting that the selection of control subjects did not seriously bias the study results. Third, we focused on only 2 SNPs in the NR5A2 gene and therefore other SNPs in this gene and other GWASs-identified SNPs remain to be replicated.

In conclusion, our case-control study found that rs3790844 in the *NR5A2* gene is associated with pancreatic cancer risk in Japanese subjects. Further exploration of genetic variation in the *NR5A2* gene and its effects on gene function could improve our understanding of pancreatic cancer pathogenesis and contribute to identifying high-risk individuals.

## Materials and Methods

### Study subjects

In this genetic case-control association study, the cases and control subjects were recruited through five participating hospitals from April 1, 2010, through May 15, 2012. The cases were eligible if they had been diagnosed with pancreatic cancer during the study period. The diagnosis was made on the basis of imaging modalities and pathology reports (if available) were reviewed for final diagnosis. Patients were excluded if they had received chemotherapy for pancreatic cancer prior to entry into the study. The control subjects were ascertained from inpatients and outpatients at each participating hospital, as well as from individuals who underwent medical checkups at one of the participating hospitals. All of the control subjects had no history of cancer. The response rate was 85% for cases and 98% for control subjects. The control subjects were frequency matched to the case patients on sex and age (within 10-year categories). As a result, 360 case patients and 400 control subjects were included in the present analysis. Approximately 90% of tumors were histologically confirmed and all tumors were adenocarcinomas. We obtained written informed consent from all of the study subjects. The Ethics Board of Aichi Medical University and the Institutional Review Board of each participating hospital approved this study. All experiments were performed in accordance with the approved guidelines.

### Data collection

We used a self-administered questionnaire to collect data on demographic characteristics, medical history, and lifestyle factors, including smoking and drinking. After giving written informed consent, the subjects provided a 7 mL venous blood sample. Genomic DNA was extracted at SRL Hachioji Laboratory using the same protocol for cases and controls. DNA samples were then stored at −30 °C until analysis.

### Genotyping assays

We selected rs3790843 and rs3790844 for genotyping because they are among the top hits in the prior GWAS. All genotyping was performed using Fluidigm SNPtype assays at the laboratory of the Aichi Cancer Center Research Institute, Nagoya, Japan. To avoid potential batch effect, the genotyping was conducted within a short period of time and there were no personnel changes. In addition, case and control samples were randomly distributed in different batches, and the laboratory staff members were blinded to the case or control status. Four quality control samples (negative control and positive controls for major homozygote, heterozygote and minor homozygote) were included in each assay, and the successful genotyping rate was 100%.

### Statistical analysis

A chi-square test was used to test the Hardy-Weinberg equilibrium (HWE) in the control subjects. Because the biological function of most SNPs has not been clearly defined, a co-dominant genomic model was assumed for SNP effects. The major allele for rs3790843 and rs3790844, which was reported in the original GWAS, served as the reference group in this study. Unconditional logistic regression models were used to estimate odds ratios (ORs) and 95% confidence intervals (CIs) for the associations between genotypes and pancreatic cancer risk. Both the crude and multivariable-adjusted ORs were presented. The multivariable model included age (continuous), sex (male or female), BMI (<20, 20–22.4, 22.5–24.9, or ≧25.0), and cigarette smoking (current, former, or never). We also conducted an analysis classifying the subjects into two groups according to BMI (<25.0 or ≧25.0) to analyze whether the genotype-pancreatic cancer association was modified by obesity. All of the tests were two-tailed, and a P value less than 0.05 was considered statistically significant. FPRP was calculated to assess whether our findings for these two SNPs were deserving of attention or “noteworthy,” based on the method proposed by Watcholder S *et al.*^25^. All of the statistical analyses were performed using SAS 9.12 (SAS Inc, USA).

## Additional Information

**How to cite this article**: Ueno, M. *et al.* Genome-wide association study-identified SNPs (rs3790844, rs3790843) in the *NR5A2* gene and risk of pancreatic cancer in Japanese. *Sci. Rep.* **5**, 17018; doi: 10.1038/srep17018 (2015).

## Supplementary Material

Supplementary Information

## Figures and Tables

**Table 1:** Selected characteristics of cases and controls.

	Cases	Controls	P
	n = 360	n = 400	
Mean age (SD)	67.8 (8.8)	64.8 (9.5)	<0.0001
Sex, n (%)			0.38
Male	215 (59.7)	226 (56.5)	
Female	145 (40.3)	174 (43.5)	
Body mass index (mean, SD)	22.9 (3.3)	22.8 (3.2)	0.42
History of Diabetes, n (%)			<0.0001
Yes	87 (24.1)	35 (8.7)	
No	269 (74.7)	362 (90.5)	
Cigarette Smoking, n (%)			<0.0001
Ever	215 (59.7)	198 (49.5)	
Never	145 (40.2)	202 (50.5)	
Number of cigarettes smoked per day (mean, SD)	20.3 (9.0)	16.2 (9.2)	0.009
Tumor stage, n(%)			
I	15 (4.2)		
II	54 (15.0)		
III	77 (21.4)		
IV	210 (58.3)		
Unknown	4 (1.1)		

**Table 2:** Associations between NR5A2 SNPs and risk of pancreatic cancer.

Gene	SNP	Genotype	Case, n	Control, n	Crude OR(95% CI)	*Multivariable-adjusted OR(95% CI)	P*
NR5A2	Rs3790843	GG	30	25	1.00	1.00	
		AG	155	157	0.82 (0.46–1.46)	0.75 (0.41–1.36)	
		AA	175	218	0.67 (0.38–1.18)	0.60 (0.33–1.08)	
		per-allele				0.78 (0.62–0.99)	0.0046
NR5A2	Rs3790844	TT	40	27	1.00	1.00	
		CT	165	163	0.68 (0.40–1.17)	0.65 (0.37–1.14)	
		CC	155	210	0.50 (0.29–0.85)	0.47 (0.27–0.83)	
		per-allele				0.70 (0.56–0.89)	0.003

*Adjusted for age, sex, body mass index, diabetes, and cigarette smoking.

**Table 3:** Associations between NR5A2 SNPs and risk of pancreatic cancer by BMI

Gene	SNP	Genotype	Case, n	Control, n	*Multivariable-adjusted OR(95% CI)
BMI<25					
NR5A2	Rs3790843	GG	25	20	1.00
		AG	119	116	0.82 (0.42–1.59)
		AA	136	177	0.62 (0.32–1.20)
	Rs3790844	TT	28	20	1.00
		CT	128	122	0.75 (0.39–1.43)
		CC	124	170	0.54 (0.28–1.03)
BMI> = 25					
NR5A2	Rs3790843	GG	5	5	1.00
		AG	36	41	0.67 (0.16–2.82)
		AA	39	41	0.51 (0.12–2.17)
	Rs3790844	TT	12	7	1.00
		CT	37	41	0.46 (0.15– 1.42)
		CC	31	40	0.31 (0.10–1.00)

*Adjusted for age, sex, body mass index, diabetes, and cigarette smoking.
