# Supplementary material for: Genome-wide association study-identified SNPs (rs3790844, rs3790843) in the NR5A2 gene and risk of pancreatic cancer in Japanese
Source: Sci Rep. 2015 Nov 23;5:17018. doi: 10.1038/srep17018 (PMC4655467; doi:10.1038/srep17018)
Supplement: Supplementary Information [file srep17018-s1.pdf]

SUPPLEMENTARY INFORMATION

Genome-wide association study-identified SNPs (rs3790844, rs3790843) in the *NR5A2* gene and risk of pancreatic cancer in Japanese

Makoto Ueno, Shinichi Ohkawa, Manabu Morimoto, Hiroshi Ishii, Masato Matsuyama, Sawako Kuruma, Naoto Egawa, Haruhisa Nakao, Mitsuru Mori, Keitaro Matsuo, Satoyo Hosono, Masanori Nojima, Kenji Wakai, Kozue Nakamura, Akiko Tamakoshi, Mami Takahashi, Kazuaki Shimada, Takeshi Nishiyama, Shogo Kikuchi, Yingsong Lin

Table S1 Results of FPRP calculations for rs3790844 based on different pre-set prior probability and ORs

		OR		
		0.6	0.7	0.8
Prior probability	0.1	0.035	0.061	0.19
	0.01	0.285	0.416	0.721
	0.001	0.801	0.878	0.963

OR: odds ratio; FPRP: false positive report probability

For rs3790844, the OR was 0.70 (95%CI:0.56-0.89) in the present study

Table S2 Results of FPRP calculations for rs3790843 based on different pre-set prior probability and ORs

		OR		
		0.6	0.7	0.8
Prior probability	0.1	0.273	0.313	0.470
	0.01	0.805	0.833	0.907
	0.001	0.977	0.981	0.990

OR: odds ratio; FPRP: false positive report probability

For rs3790843, the OR was 0.78 (95%CI: 0.62-0.99) in the present study
